# Enteral nutritional support for an elderly critically ill patient with gastroesophageal reflux disease and situs inversus totalis: a case report

**DOI:** 10.3389/fnut.2025.1514980

**Published:** 2025-02-26

**Authors:** Yuanyuan Chen, MengLin Ma, Xiaoqing Huang, Jia Wan, Haiyan Huang

**Affiliations:** Department of Critical Care Medicine, Union Hospital, Tongji Medical College, Huazhong University of Science and Technology, Wuhan, China

**Keywords:** situs inversus totalis, gastroesophageal reflux disease, malnutrition, enteral nutrition, case report

## Abstract

Herein, we present a case of serious protein-energy malnutrition in an elderly critically ill patient with situs inversus totalis. It was difficult to implement enteral nutrition in this patient for more than 2 months of hospitalisation in another hospital, and we applied electromagnetic navigation guidance to implement enteral nutrition after successful placement of nasojejunal tubes. We reviewed the management of enteral nutrition support.

## 1 Introduction

Gastroesophageal reflux disease (GERD), characterized by the retrograde flow of gastric contents into the esophagus due to dysfunctional gastroesophageal junction structure and function in adults (1, 2). The primary manifestations include a sensation of burning behind the sternum or regurgitation of gastric contents into the throat or mouth, non-cardiac chest discomfort, and extra-esophageal symptoms such as coughing, throat irritation, and voice hoarseness (3, 4). The incidence of GERD in adults is 13.98%, escalating with advancing age (5). GERD may result in complications such as esophagitis, esophageal stricture, Barrett’s esophagus, upper gastrointestinal bleeding (1), leading to insufficient food consumption and impaired nutrient absorption in the gastrointestinal tract, consequently resulting in malnutrition. Malnutrition can be classified into moderate and severe categories based on three anthropometric criteria (unintentional weight loss, low BMI, and reduced muscle mass) as well as two etiological diagnostic criteria (decreased food intake or absorption, disease, or inflammation) (6). Among European countries, the incidence of malnutrition in patients aged ≥65 years was 28% (7), while it is higher among hospitalized elderly people aged 70 years and older in China, with a prevalence of 25.4 to 32.6% (8). Protein-energy malnutrition (PEM) is a common form of nutritional deficiency in the elderly, characterized by inadequate intake of protein and/or energy to meet the body’s nutritional requirements, which can detrimentally affect immune function, diminish quality of life, and increase mortality risk (9) among the elderly, and negatively affects their physical and mental health. With the aging of the population, there is an increase in the number of elderly patients who are particularly susceptible to malnutrition due to their compromised health status (10, 11).

For critically ill elderly patients, enteral nutrition is considered the preferred method of nutritional support (12), as it contributes to the maintenance of gastrointestinal mucosal integrity and reduces the risk of infection. The primary routes of enteral nutrition administration are oral, nasogastric tube, nasojejunal tube, gastrostomy, and jejunostomy (13, 14). Short-term enteral nutrition support can be provided via nasogastric and nasojejunal tubes. A nasogastric tube is appropriate for patients without upper gastrointestinal tract obstruction and with intact gastrointestinal tract function who have received enteral nutrition for less than 4 weeks (13); however, complications such as nasopharyngeal injuries, gastroesophageal reflux, and aspiration may occur. Nasojejunal tube feeding is indicated for patients with impaired gastric emptying or unsuitability for intragastric feeding, significantly reducing the risk of aspiration, minimizing nutritional interruption, and decreasing the incidence of aspiration pneumonia, while providing effective nutritional support to critically ill patients (12).

The primary methods of nasojejunal tube placement include endoscopic guidance, X-ray guidance, ultrasound-assisted, electromagnetic guidance, and blind insertion. Blind insertion is characterized by ease of operation, cost-effectiveness, and timely performance; however, the success rate of tube placement is low. Ultrasound-assisted catheterization can be conducted at the bedside and is expeditious, although visualization is somewhat limited. Electromagnetic guidance is suitable for bedside procedures, particularly for critically ill patients, but resource availability must be considered. Endoscopic and X-ray guided catheterization are effective methods, but they are dependent on specialized equipment and physicians, cannot be performed promptly, and are associated with higher costs (12). Gastrostomy and jejunostomy are more appropriate for patients requiring long-term enteral nutritional support. Gastrostomy is suitable for patients with feeding disorders above the spout due to various etiologies, while jejunostomy is indicated for patients with significant gastroesophageal reflux, high aspiration risk, impaired gastric emptying, and those who have undergone major abdominal surgeries and gastrectomy (13). However, these two techniques may lead to complications such as infections, hemorrhage, tube displacement, or prolapse.

Situs inversus totalis (SIT) is a congenital malformation attributed to chromosomal abnormalities, characterized by a complete mirror-image transposition of the internal organs in the chest and abdomen, with an incidence rate ranging from 0.005 to 0.00125% (15). SIT may present challenges during diagnosis and treatment due to the mirror effect. For instance, during gastroduodenoscopy, the operative direction is contrary to that of the routine procedure. Interventional treatment necessitates adjustment of patient position, healthcare personnel positioning, and equipment configuration. Surgical procedures require comprehensive assessment of anatomical location and implementation of individualized surgical plans (16).

Herein, we report a case of serious protein-energy malnutrition in an elderly critically ill patient with situs inversus totalis and share strategies for implementing enteral nutritional support for the patient.

## 2 Case report

A 86-year-old male patient was admitted to the hospital on April 23, 2024, due to respiratory failure resulting from severe pneumonia. His medical history included gastroesophageal reflux disease, Parkinson’s disease, and vascular dementia. Two months prior, the patient underwent tracheotomy at a local hospital; however, extubation of the tracheostomy tube proved challenging due to persistent coughing and sputum with dyspnea. Concurrently, the patient received continuous renal replacement therapy for hepatic and renal failure. During hospitalization, multiple attempts were made to place a nasojejunal tube; however, the tip of the tube was briefly dislodged into the gastric lumen, complicating the implementation of enteral nutrition.

**Figure 1:**
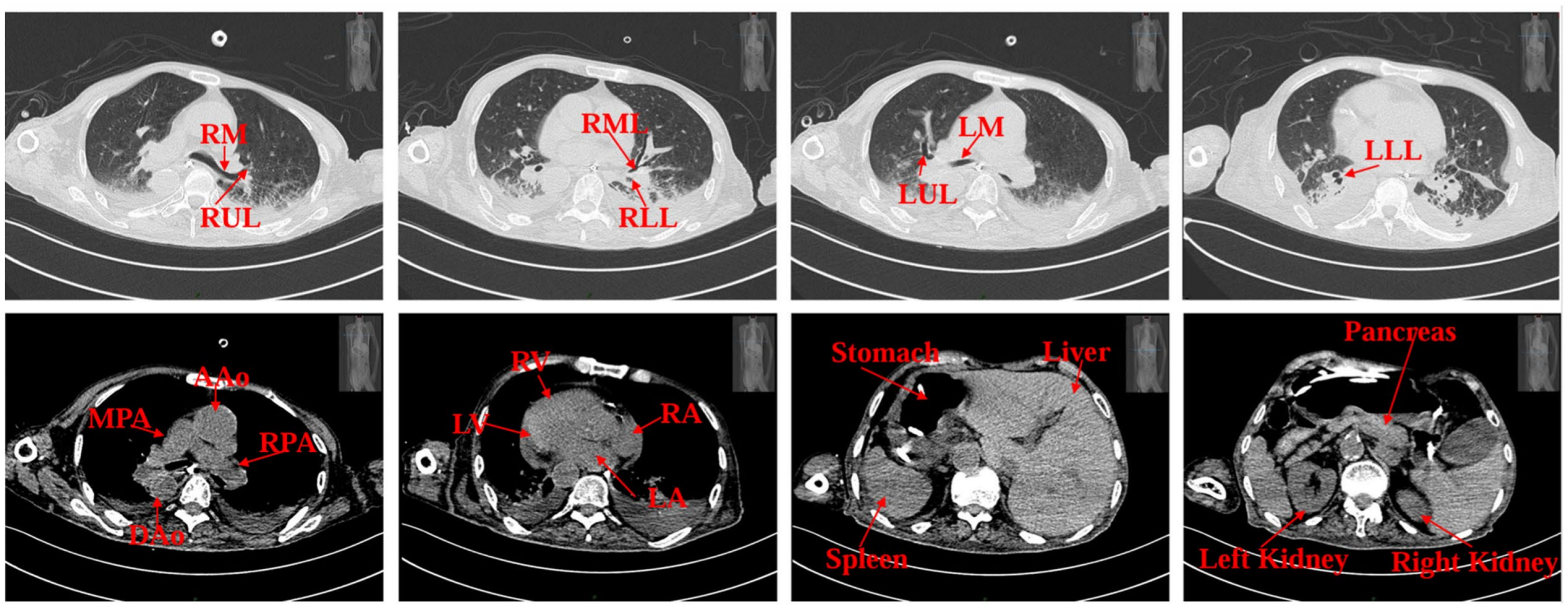
Patient’s CT scan images on admission. RM (right main bronchus), RUL (right upper lobe of lung), RML (right middle lobe of lung), RLL (right lower lobe of lung), LM (left main bronchus), LUL (left upper lobe of lung), LLL (left lower lobe of lung), MPA (main pulmonary artery), RPA (right pulmonary artery), AAo (ascending aorta), DAo (descending aorta), LV (left ventricle), LA (left atrium), RV (right ventricle), RA (right atrium).

Upon admission, the patient weighed 35 kg and stood 160 cm tall, resulting in a body mass index (BMI) of 13.67 kg/m^2^. Computed tomography scans of the cranium, abdomen, pelvis, and lungs suggested situs inversus totalis (Figure 1) with soft foci in the right temporo-occipital lobe, but without any apparent signs of obstruction in the gastrointestinal tract. Ultrasonography revealed a mirrored right-sided heart and partial intramuscular vein thrombosis in the left calf. Laboratory examinations yielded the following results: platelet count of 44 × 10^9^/L, albumin level of 31.1 g/L, prealbumin level of 0.135 g/L, hemoglobin concentration of 76 g/L, serum phosphorus level of 0.64 mmol/L, serum potassium level of 2.35 mmol/L, serum sodium level of 132.3 mmol/L and serum calcium level of 1.87 mmol/L. Nutritional risk screening, conducted using the Nutrition Risk Screening 2002 (NRS 2002) tool, resulted in a score of 7, indicating a risk of malnutrition. Due to the patient’s prolonged bedridden state and the challenges associated with implementing enteral nutrition at an external facility, a consultation with the gastrointestinal surgery department was urgently requested on the day of admission, which recommended jejunostomy. However, the patient’s family declined jejunostomy, considering the patient’s advanced age and associated surgical risk. Subsequently, a nurse-led, case-based nutritional support treatment team was established, comprising a critical care nurse leader, critical care physician, critical care nutrition nurse specialist, and specialized nurses from the nutrition department.

**Figure 2:**
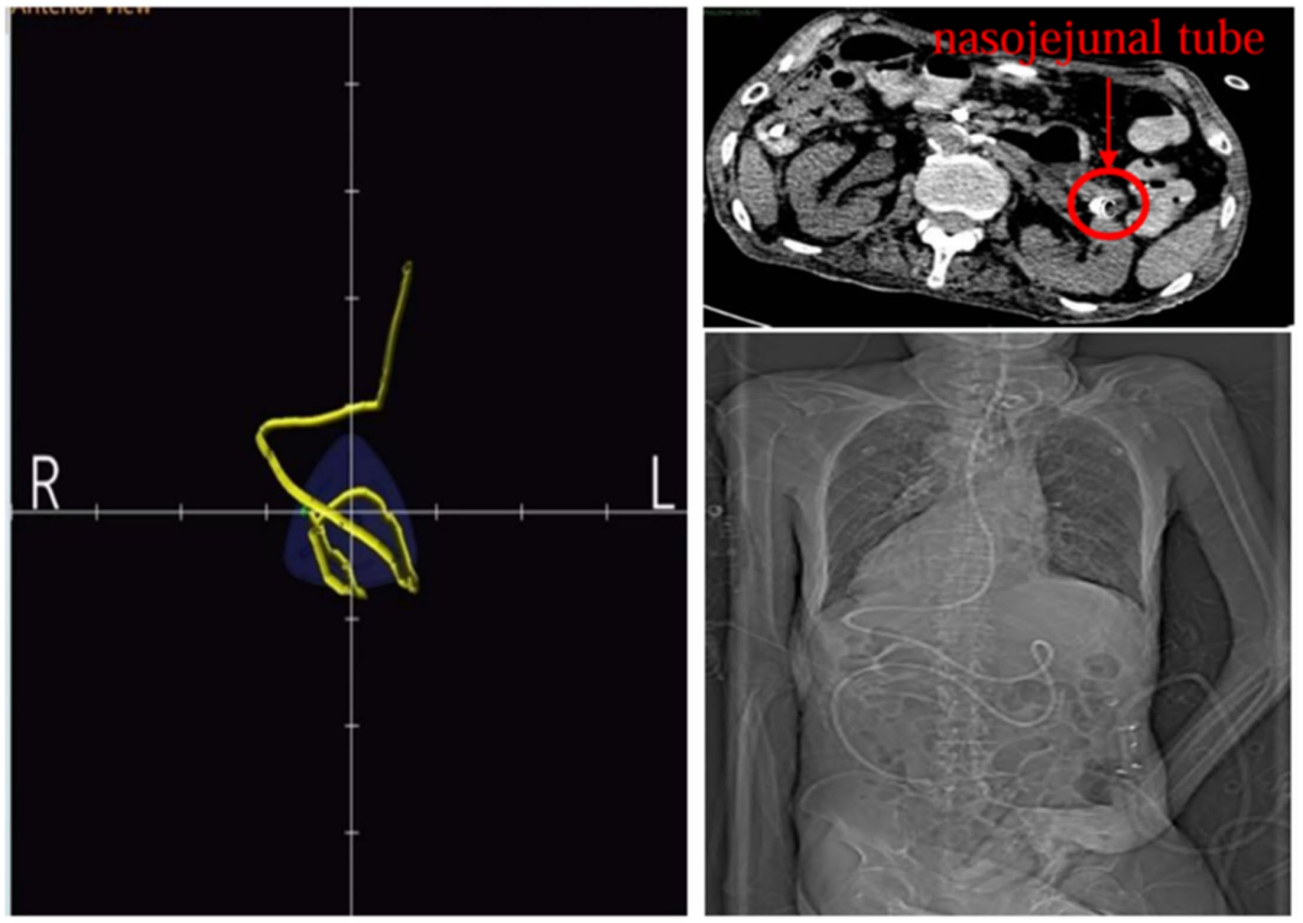
Location of the tip of the nasojejunal tube after first tube placement.

**Figure 3:**
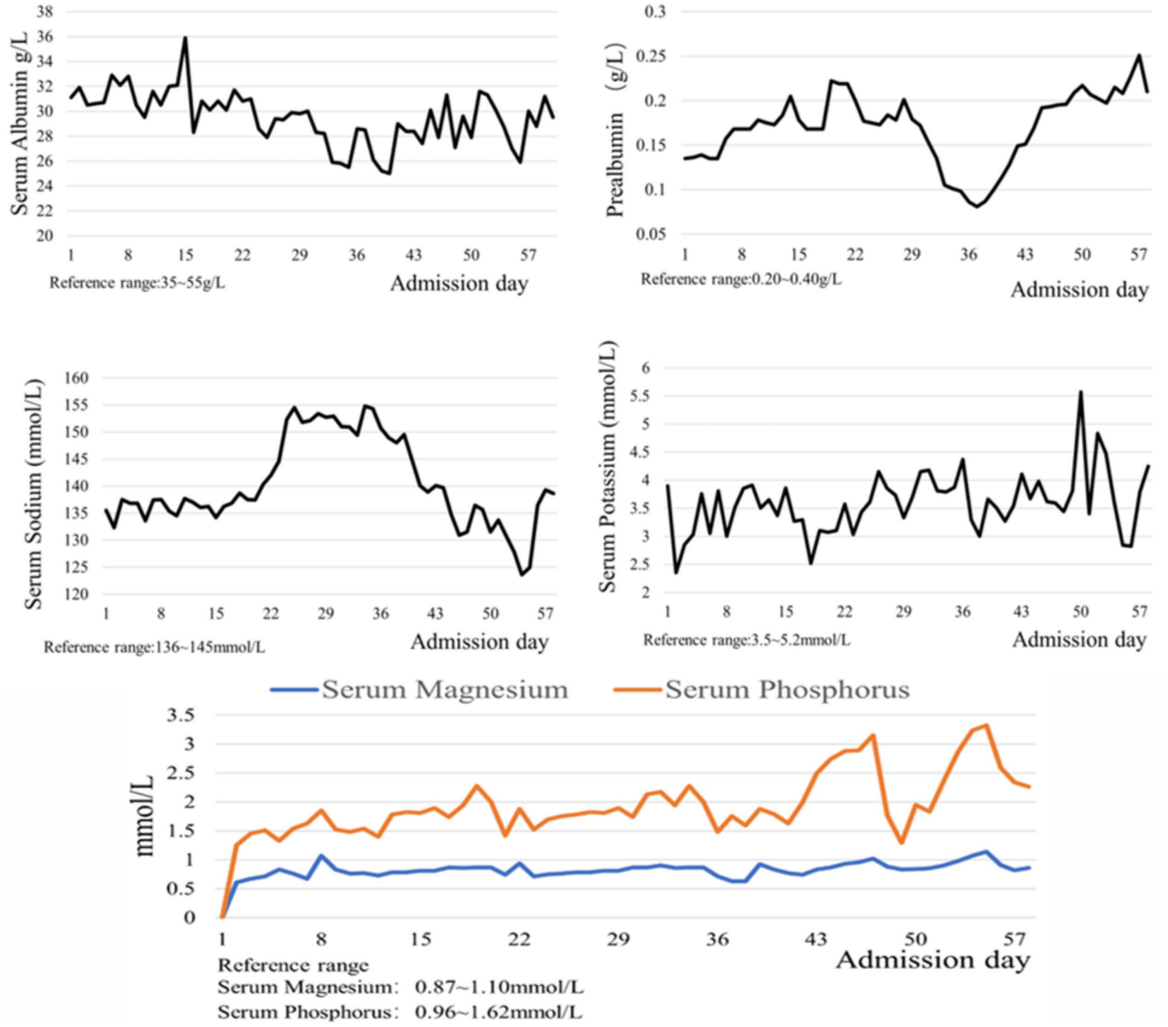
The trends of laboratory examinations results.

The Acute Gastrointestinal Injury Ultrasonography Score (AGIUS) (17) was used to assess the extent of gastrointestinal function impairment in the patient. The results indicated a 3.5 cm diameter of the intestinal tube, no alterations in the intestinal folds, a 3 mm thickness of the intestinal wall, and a peristalsis rate of three contractions per minute, yielding a score of 2. Acute Gastrointestinal Injury (AGI) was classified as grade 1. Given the patient’s history of gastroesophageal reflux, gastric ulcer with bleeding, and high aspiration risk, enteral nutrition was administered via a nasojejunal tube. Compared to endoscopic-guided tube placement, electromagnetic navigation-guided nasojejunal tubes offer cost reduction (18), feasibility at the bedside, and do not require specialist intervention (12). Furthermore, there is little difference between the two methods regarding time, safety, and success rates (18). Considering the patient’s situs inversus totalis, serious status, and need for nasojejunal tube replacement, the team was elected for electromagnetic navigation to place a single-lumen nasojejunal tube at the bedside. On the third day of admission, a nasojejunal tube was placed for the first time using reverse thinking. Abdominal computed tomography after initial placement showed that the tip of the nasojejunal tube was located in the jejunum (Figure 2). Subsequently, enteral nutritional support was initiated with food for special medical purposes (FSMP). Nutritional support was gradually transitioned from total parenteral nutrition to total enteral nutrition through an early nourishing enteral nutrition program. According to practical guidelines on nutritional therapy in critical geriatrics in China (14), the nutrition support energy goal was set at 875 ~ 1050 kcal·d^−1^, and the protein goal was established at 42 ~ 70 g·d^−1^. A total nutritional formula for special purposes was selected, and the implementation of specific enteral nutrition is detailed in Table 1. There were no complications, such as vomiting, bloating, diarrhea, or refeeding syndrome. The process resulted in nasojejunal tube replacement on May 24 and June 21, respectively, as it was time for replacement. The trends in the laboratory examination results are illustrated in Figure 3.

**Table 1:** Implementation of enteral nutrition.

	Enteral nutrition formulations	Energy density	Feeding rate	Feeding time	Energy intake	Protein intake
1st day						
2nd day						
3rd day	FSMP 125 mL and water 125 mL	0.6 kcaL/mL	9 kcal/h	16.7 h	150 kcal/d	5 g/d
4th day	FSMP 250 mL and water 250 mL	0.6 kcaL/mL	15 kcal/h	20 h	300 kcal/d	10 g/d
5th day	FSMP 500 mL and water 500 mL	0.6 kcaL/mL	30 kcal/h	20 h	600 kcal/d	20 g/d
6th day	FSMP 1000 mL	1.2 kcaL/mL	60 kcal/h	20 h	1,200 kcal/d	40 g/d
7th day	FSMP 1000 mL	1.2 kcaL/mL	72 kcal/h	16.7 h	1,200 kcal/d	40 g/d

## 3 Discussion

SIT is a rare anatomical variant in which complete thoracic and abdominal visceral inversions involve 180-degree rotation of all internal organs, including the heart, liver, spleen, stomach, and intestines (19). The incidence of SIT is extremely low, making the clinical investigation and development of therapeutic strategies for affected patients particularly challenging. Disorders of esophageal and gastric motility are considered key factors contributing to pathological reflux in GERD, including insufficient contraction of the lower esophageal sphincter, decreased esophageal contouring ability, and delayed gastric emptying, all of which may lead to reflux of gastric acid and contents into the esophagus (20), resulting in typical GERD symptoms, such as reflux and heartburn. It is crucial to consider not only the anatomical variations but also the potential challenges associated with delayed gastric emptying when placing nasojejunal tubes in patients with SIT. These factors are likely to increase the difficulty and complexity of the procedure. Additionally, the patient had Parkinson’s disease with impaired swallowing, which increased the risk of aspiration during nasojejunal tube placement.

**Figure 4:**
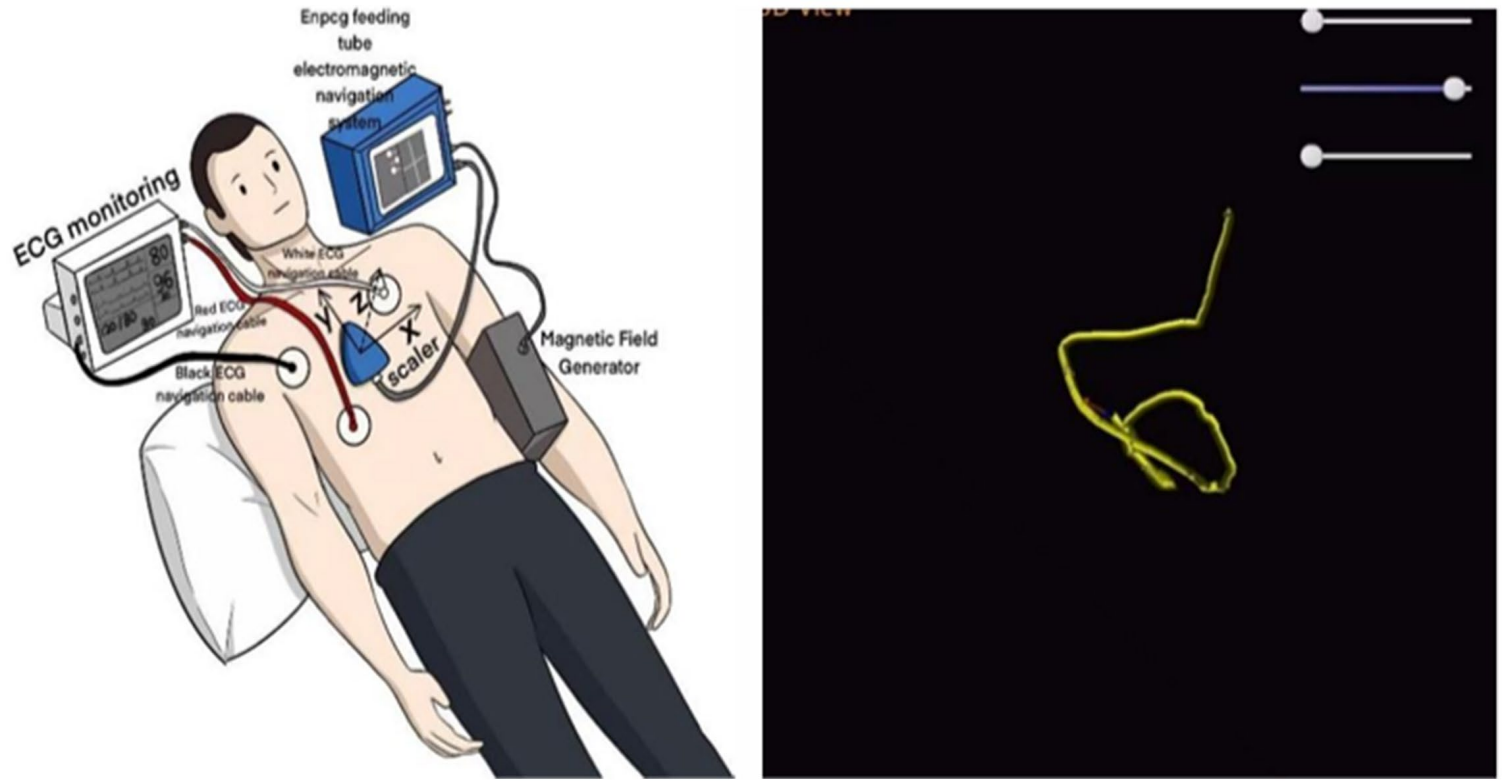
Use of electromagnetic navigation for inserting the nasojejunal tube.

In the present case, tube placement was performed by an intensive care nutrition specialist nurse trained in electromagnetic navigation. Prior to the procedure, 10 mg of metoclopramide was administered intravenously to enhance gastric emptying, and once it was confirmed that the nasojejunal tube was positioned in the stomach, 300 mL of air was introduced to facilitate the opening of the pyloric orifice. When the nasojejunal tube was coiled in the stomach for the third time during placement, the operator gradually increased the number of guidewires within the tube until three guidewires were in place. The tube was then delivered slowly and uniformly to incrementally increase catheter rigidity. Upon reaching the pyloric portal, the tip of the nasojejunal tube encounters resistance and cannot pass smoothly, even with a guidewire in place. To reduce the turning radius, the operator withdrew the three guidewires by 5 cm while relying on intestinal peristalsis to navigate through the pyloric portal. Subsequently, the tip of the nasojejunal tube was positioned in the jejunum by adjusting the X-, Y-, and Z-axes (Figure 4). Abdominal computed tomography after the initial tube placement revealed that the tip of the nasojejunal tube was located in the jejunum, indicating successful placement. Using the same technique, the subsequent two nasojejunal tube replacements were completed without complications, such as fluctuations in blood pressure, temporary decreases in oxygen saturation, pneumothorax, gastrointestinal bleeding, or perforation. In this instance, the tip of the nasojejunal tube was dislodged into the gastric lumen at another hospital; however, this did not occur during the later stages of enteral nutrition implementation in our facility. A possible explanation for this discrepancy may be the use of different materials for the nasojejunal tubes. This case demonstrates that electromagnetic navigation can effectively guide the placement of nasojejunal tubes in patients who experience difficulties with tube placement or who have unstable circulation and cannot undergo enterostomy or endoscopic procedures, particularly in those who are bedridden for extended periods.

Refeeding Syndrome (RFS) is a metabolic disorder that occurs when the metabolic state transitions from catabolism to anabolism in chronically starved or malnourished patients receiving nutritional support. Its pathogenesis is primarily associated with increased insulin secretion, the transfer of electrolytes to cells, and the enhancement of anabolism. In malnourished patients, the body is depleted of phosphorus, potassium, magnesium, vitamin B1, and other nutrient reserves, although serum electrolyte levels may remain within normal ranges (21–23). Upon initiation of nutritional therapy, particularly when carbohydrate intake is increased, insulin secretion is restored, promoting the intracellular movement of potassium, phosphorus, and magnesium, leading to hypokalemia, hypophosphatemia, and hypomagnesemia. Concurrently, enhanced anabolic metabolism results in significant vitamin B1 depletion, ultimately precipitating refeeding syndrome. The prevalence of refeeding syndrome in ICU patients ranged from 10.3 to 100% (24). A study has indicated that (22) individualized nutritional support and fluid management strategies should be implemented to prevent refeeding syndrome, based on the patient’s risk level. These strategies encompass a gradual increase in energy supply, electrolyte and vitamin supplementation, and close monitoring. According to the screening criteria for refeeding syndrome risk factors outlined in the Clinical Application Guidelines for Parenteral Enteral Nutrition in Adult Patients in China (13), we assessed that this case, presenting with severe protein-energy malnutrition, a BMI of 13.67 kg/m2, and a serum phosphorus level of 0.64 mmol/L at admission, was at high risk for refeeding syndrome. An observational study (25) reported that the severity of refeeding syndrome was not correlated with energy prescription, prophylactic electrolyte administration, or micronutrient supplementation. In critically ill patients, the implementation of energy restriction to prevent refeeding syndrome remains controversial (26). However, considering that high-energy enteral nutrition intake, which increases insulin secretion, may further reduce blood phosphorus levels. A guide to enteral nutrition in intensive care units (27) recommends that energy and protein should be supplied at low levels initially, with gradual increases to the target amount. During the implementation of nutritional support for this patient, enteral nutrition was initiated at low energy levels and gradually increased. Concurrently, liver and kidney function and metabolic indices such as blood glucose, blood lipids, and electrolytes were monitored daily, while intravenous phosphorus supplementation with compound potassium hydrogen phosphate was administered according to the test results. The patient was discharged from the hospital with a blood phosphorus level of 1.40 mmol/L, and no refeeding syndrome occurred.

Enteral Feeding Intolerance (EFI) is defined as the cessation or reduction of enteral nutrition infusion due to gastrointestinal dysfunction (28). The complex pathophysiology of EFI encompasses autonomic nervous system dysfunction, hormonal dysregulation, pharmacological effects, electrolyte and blood glucose imbalances, inflammatory responses, and intestinal microbiota dysbiosis, resulting in gastrointestinal motility disorders characterized by elevated gastric residual volume, emesis, diarrhea, abdominal distension, and increased intra-abdominal pressure (28). The prevalence of feeding intolerance in critically ill patients ranges from 29.17 to 60.47% (29). Age exceeding 60 years, gastrointestinal disease, low Glasgow Coma Scale score, hypokalemia, medication use, and mechanical ventilation are identified risk factors for EFI while enteral nutrition preparations containing dietary fiber serve as protective factors (29, 30). The patient, presenting with advanced age, gastroesophageal reflux disease, hypokalemia, and vascular dementia, was at high risk of feeding intolerance. In this case, a nurse-led multidisciplinary case nutrition support team implemented enteral nutrition support via a nasojejunal tube, utilizing an enteral nutrition preparation containing soluble dietary fiber, administered at an initial rate of 15 mL/h. On the 13th day of enteral nutrition implementation, the patient’s fecal characteristics transitioned from yellow paste stool to yellow dilute stool. Following team deliberation, pharmacological interventions to enhance gastrointestinal motility and intestinal flora balance were introduced to mitigate feeding intolerance. Enteral nutrition was administered for 58 days, during which no interruptions or complications such as emesis, diarrhea, abdominal distension, or constipation were observed. The enteral nutrition support provided to this patient aligned with the best evidence summary for enteral nutrition intolerance in critically ill patients (31), which advocates for a multidisciplinary team approach in formulating and implementing enteral nutrition plans for critically ill patients, recommends the inclusion of dietary fiber in enteral nutrition preparations, and suggests initiating enteral nutrition at a rate of 10–30 mL/h to prevent EFI.

The rate of nasojejunal tube occlusion is 4–8% (32), which affects the implementation of enteral nutrition. To prevent obstruction of the nasojejunal tube, after successfully placing the nasojejunal tube, we placed a nasogastric tube for nasal drug administration to avoid blockage caused by the reaction between the drug and nutritional preparation. During the continuous infusion of enteral nutrition by the nutrition pump, warm water was used to flush the nasojejunal tube at regular intervals of 4 h. In the present case, there was no blockage of the nasojejunal tube.

In this case, a nurse-led multidisciplinary team that implemented enteral nutrition support therapy was successful. The establishment of a multidisciplinary team is recommended for enteral nutrition support in critically ill patients (31). Compared to non-nurse-driven protocols, nurse-driven enteral nutrition protocols may enhance adherence, reduce feeding interruptions, and facilitate the implementation of individualized nutritional regimens among critically ill adult patients (33). Nurses play an important role in assessing and preventing complications associated with enteral nutrition (34). However, a significant percentage of nurses possess inadequate knowledge of enteral nutrition and exhibit poor clinical practice (35). Therefore, it is essential to enhance their knowledge and skills in enteral nutrition through regular in-service training and continuing education programs. Standard operating procedures and protocols for enteral nutrition should also be developed and implemented in hospitals, enabling nurses to follow clear guidelines in their practice, thereby improving the quality of care and patient safety.

This study had several limitations. During hospitalization, the patient’s weight was not regularly measured because of the absence of weight-measurement capability in the unit’s beds. Although the patient successfully received enteral nutrition, the team did not adjust the nutritional regimen to account for continuous renal replacement therapy. Additionally, the patient was discharged unexpectedly, preventing further monitoring of nutritional status.

## 4 Conclusion

This case report elucidates the efficacious management of severe protein-energy malnutrition in an 86-year-old critically ill patient with gastroesophageal reflux disease and situs inversus totalis. The distinctive anatomical challenges presented by situs inversus totalis, in conjunction with the patient’s comorbidities, necessitated a tailored approach to enteral nutrition support. Through the utilization of electromagnetic navigation-guided nasojejunal tube placement, we were able to surmount the difficulties associated with conventional tube placement methods, ensuring effective and sustained enteral nutrition delivery for 58 days without complications. The multidisciplinary team approach, spearheaded by nursing specialists, was instrumental in addressing the patient’s complex needs, including the prevention of refeeding syndrome and enteral feeding intolerance. This case highlights the importance of personalized enteral nutrition support protocols and the efficacy of a collaborative, multidisciplinary approach in the management of critically ill geriatric patients presenting with complex anatomical and nutritional challenges.

## Data Availability

The raw data supporting the conclusions of this article will be made available by the authors, without undue reservation.
